# Supplementary material for: Characterization of 3D Organotypic Culture of Mouse Adipose-Derived Stem Cells
Source: Int J Mol Sci. 2024 Apr 1;25(7):3931. doi: 10.3390/ijms25073931 (PMC11011465; doi:10.3390/ijms25073931)
Supplement: Supplementary file 1 [file ijms-25-03931-s001.zip › ijms-2860309-supplementary.pdf]

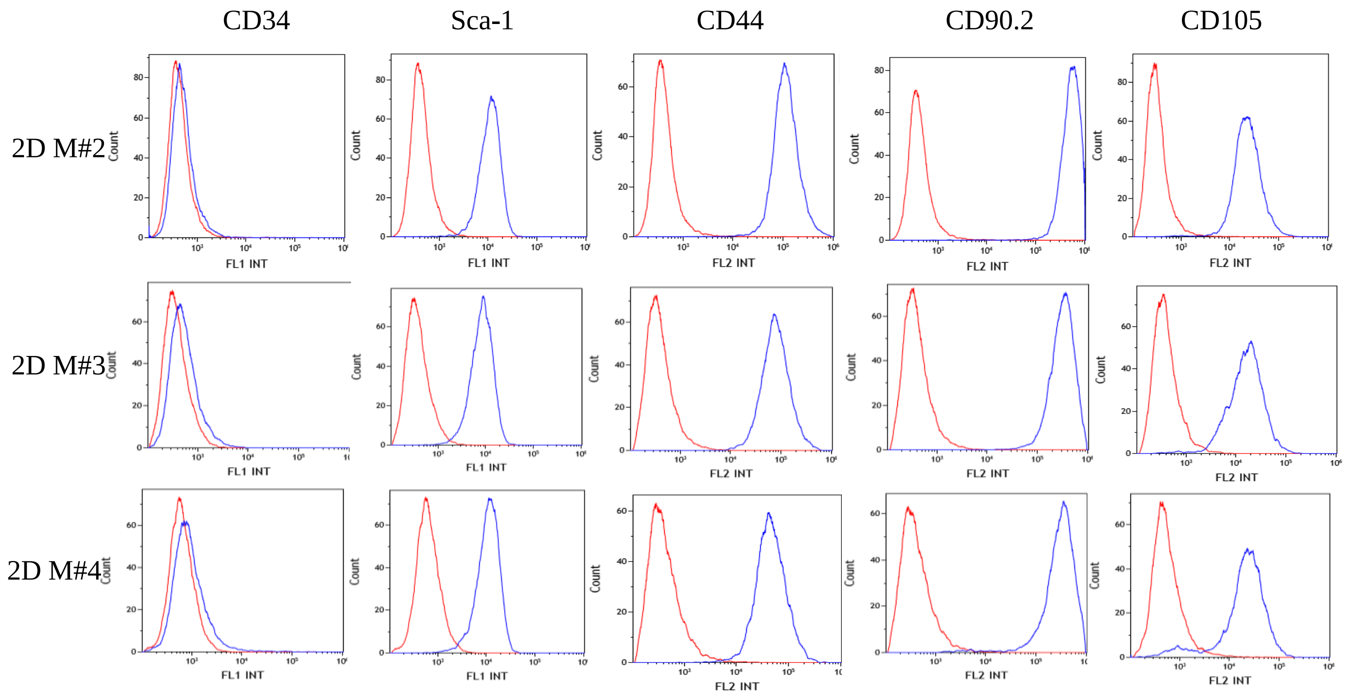


Supplementary Figure S1A

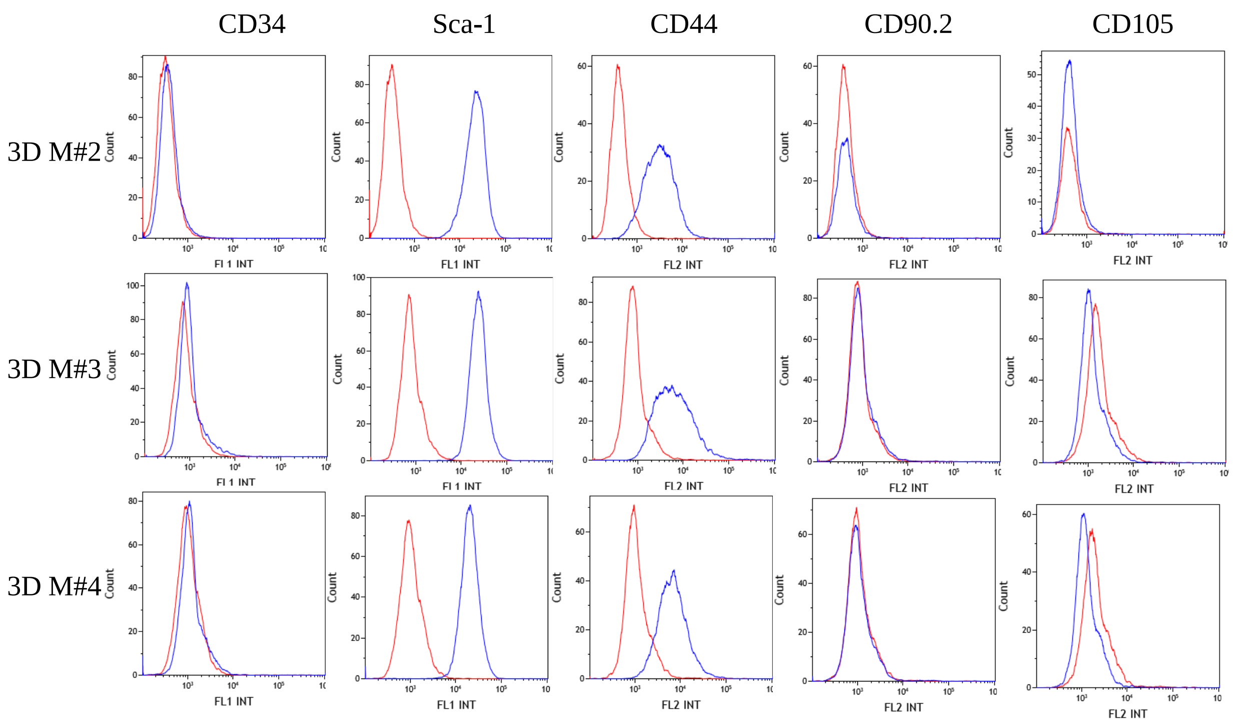


Supplementary Figure S1B

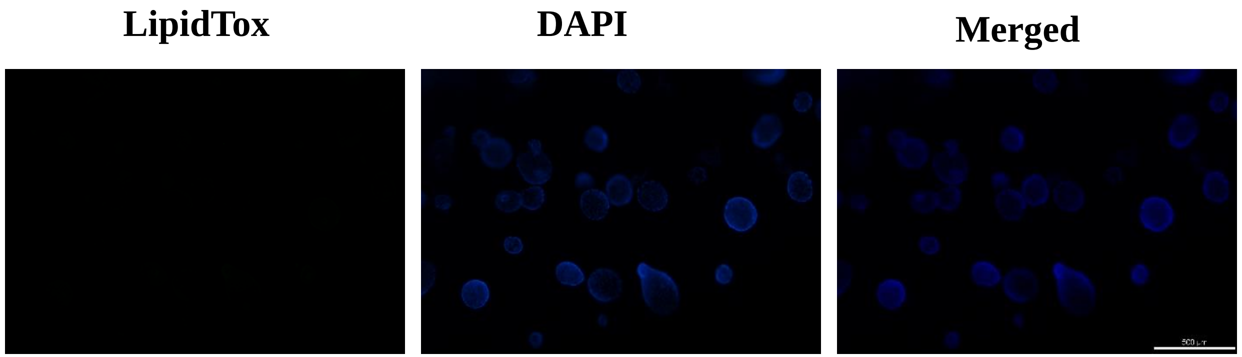


Supplementary Figure S2

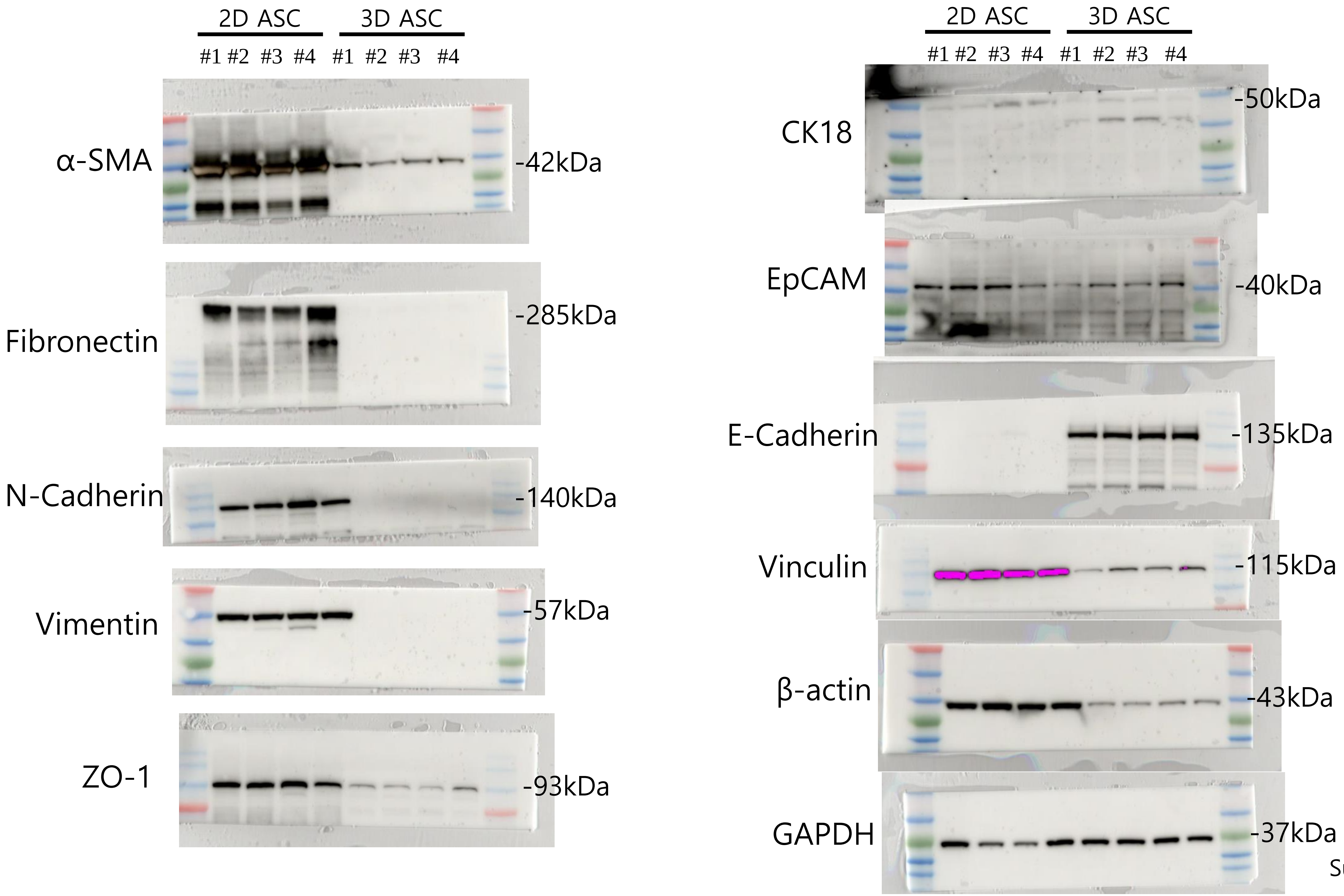


Supplementary Figure S3

Supplementary legends

Figure S1. Additional flow cytometry analysis results for ADSCs from mice #2, #3, and #4.
(A) Histograms for 2D-cultured ADSCs.
(B) Histograms for 3D-cultured ADSCs.

Figure S2. Analysis of adipogenic differentiation using staining methods.
The 3D-cultured ADSCs were stained with LipidTox, a fluorescent stain for fat, and DAPI was used for nuclear staining.

Figure S3. Original blots/gels are presented
